# Semaglutide treatment for obesity in teenagers: a plain language summary of the STEP TEENS research study

**DOI:** 10.2217/cer-2022-0187

**Published:** 2022-12-19

**Authors:** Daniel Weghuber, Klaus Boberg, Dan Hesse, Ole K Jeppesen, Rasmus Sørrig, Aaron S Kelly, for the STEP TEENS Investigators

**Affiliations:** 1 Department of Pediatrics, Paracelsus Medical University, Salzburg, Austria; 2 Novo Nordisk A/S, Søborg, Denmark; 3 Department of Pediatrics & Center for Pediatric Obesity Medicine, University of Minnesota Medical School, Minneapolis, MN, USA

**Keywords:** BMI, body mass index, obesity, plain language summary, semaglutide, STEP TEENS, teenagers, weight loss

## Abstract

**What is this summary about?:**

This is a plain language summary of the STEP TEENS research study, which was originally published in the *New England Journal of Medicine*. As more teenagers are living with obesity than ever before, researchers are searching for new treatments. This was the first study looking at how well the medicine semaglutide works as a treatment for obesity in teenagers.

**What were the results?:**

In this study, researchers looked at the effect of semaglutide on body mass index (BMI) and weight loss compared to a dummy medicine (placebo). A 17% decrease in BMI was reported for teenagers treated with semaglutide compared with placebo. For weight loss, an 18 kg decrease was seen when comparing semaglutide with placebo. Researchers found that there were more teenagers who had weight loss of 5%, 10%, 15%, and 20% or more in the group given semaglutide compared with the group given placebo. Improvements were also seen with semaglutide treatment for some risk factors for other diseases caused by obesity. Semaglutide was generally well tolerated by the teenagers with obesity in this study, and serious medication side effects did not happen very often.

**What do the results mean?:**

The results from this study showed that there were no safety issues with semaglutide in teenagers with obesity, and that semaglutide can be used to help them lose weight.

**Clinical Trial Registration**: NCT04102189 (ClinicalTrials.gov)

To read the full Plain Language Summary of this article, click here to view the PDF.

Link to original article here

